# Identification of Metabolites in Muscles of Lueyang Black-Bone Chickens: A Comparative Analysis of Caged and Cage-Free Rearing Modes Using Untargeted Metabolomic Techniques

**DOI:** 10.3390/ani14142041

**Published:** 2024-07-12

**Authors:** Ling Wang, Jie Gao, Guojin Li, Jia Cheng, Guoqiang Yuan, Tao Zhang, Wenxian Zeng, Hongzhao Lu

**Affiliations:** 1 School of Biological Science and Engineering, Shaanxi University of Technology, Hanzhong 723001, China; 2 Shaanxi University Engineering Research Center of Quality Improvement and Safety Control of Qinba Special Meat Products, Hanzhong 723001, China; 3 Qinba Mountain Area Collaborative Innovation Center of Bioresources Comprehensive Development, Shaanxi University of Technology, Hanzhong 723001, China; 4 Qinba State Key Laboratory of Biological Resources and Ecological Environment, Shaanxi University of Technology, Hanzhong 723001, China; 5 Shaanxi Baiweiyuan Network Technology Company, Hanzhong 724300, China

**Keywords:** black-bone chicken, rearing system, meat quality, metabolomics

## Abstract

**Simple Summary:**

The Lueyang black-bone chicken is a vital poultry resource originating from China. This study aimed to investigate the effects of different rearing systems on the meat quality of Lueyang black-bone chickens via untargeted metabolomics. Compared with caged rearing, cage-free rearing significantly reduced chicken carcass yield and increased the shear force of leg muscles. In addition, the cage-free rearing mode promoted the accumulation of nutrients, such as phospholipids, unsaturated fatty acids, glutamate, and adenosin 3′-monophosphate. Moreover, the cage-free rearing system also enhanced the accumulation of volatile organic compounds, such as aldehydes, alcohols, and ketones, which greatly contribute to the meat flavor. In summary, this study provides a theoretical basis for cage-free rearing as a better rearing mode for Lueyang black-bone chickens.

**Abstract:**

The Lueyang black-bone chicken is a specific native chicken strain in China. This study aimed to investigate the effects of different rearing systems on the meat quality of Lueyang black-bone chickens. Six hundred Lueyang black-bone hens were randomly divided into two groups at 7 weeks of age and raised in cage and cage-free systems for 20 weeks. The carcass yield, meat quality, and total metabolites were measured in both the leg and breast muscles. By comparison, the carcass yield of hens in the cage-free (CF) group (1.26 ± 0.09 kg) was significantly lower than that in the caged rearing (CR) group (1.52 ± 0.15 kg). However, the shear force of leg muscles in the CF group (27.98 ± 2.43 N) was significantly greater than that in the CR group (24.15 ± 1.93 N). In addition, six samples from each group were randomly selected and their metabolites were detected by the non-targeted metabolomics technique. Among these metabolites, 408 and 354 significantly differentially abundant metabolites were identified in breast and leg muscles, which were mainly involved in glycerophospholipid metabolism, unsaturated fatty acid biosynthesis, arginine and proline metabolism, and nucleotide metabolism. We found that the levels of 19 phospholipids, mainly phosphatidylcholines and lysophosphatidylcholines, were significantly greater in the CF group than in the CR group. Additionally, the contents of eight unsaturated fatty acids, linoleic acid, and linolenic acid were dramatically greater in the CF group than in the caged group. The accumulation of 4-hydroxy-proline, glutamate, and adenosine 3′-monophosphate (AMP) was enhanced in the CF group. Moreover, many more volatile organic compounds were identified in the muscles of the cage-free group, enhancing the flavor of the chicken meat. In conclusion, the cage-free rearing mode facilitates the accumulation of nutrients and flavor substances in the chicken meat and is a better rearing system for Lueyang black-bone chickens.

## 1. Introduction

The black-bone chicken is a vital poultry resource originating from China. A number of body parts of black-bone chickens appear black in color, mainly in the skin, bone, and meat [1]. Specifically, black-bone chicken meat is rich in nutritional substances, including phospholipids, polypeptides, amino acids, vitamins, and melanin [2]. A variety of characteristic peptides in black-bone chicken meat have immunomodulatory effects due to significantly enhancing the proliferation of T and B lymphocytes and the phagocytosis of macrophages [3]. Melanin has a wide range of biochemical activities, such as antioxidation, anti-inflammatory, and immunoregulatory functions [4,5]. It is important to mention that black-bone chicken meat has certain medicinal value. Studies have shown that black-bone chicken meat contains many antioxidative peptides (such as Glu-Pro-Asp-Arg-Tyr and carnosine), which can reduce the occurrence of numerous diseases, such as atherosclerosis, diabetes, neurodegenerative disorders, and aging in the human body, by radical scavenging, reducing, and metal ion chelating activity [6,7]. Therefore, black-bone chickens are considered to have medicinal properties and are an important component of medicinal diets [8].

Rearing systems have significant effects on chicken welfare, growth performance, carcass traits, meat quality, immune status, and stress resistance [9,10]. Currently, caged and cage-free rearing systems are the primary methods used for commercial chicken raising. The benefit of the caged rearing system is that it can maximize the use of confined space and resources to improve the efficiency of production [11]. Caged rearing for chickens is the dominant commercial raising system and has the advantages of superior feed efficiency and growth performance [12]. However, chickens can also experience considerable stress in caged rearing mode [13], which may cause negative physiological and behavioral responses. In contrast, the cage-free rearing mode has either no restrictions or outdoor activity space for chickens. So, cage-free rearing mode is considered to be natural and environmentally friendly, while also improving the comfort and welfare of chickens [14]. A few previous studies revealed that the cage-free group performed better in terms of nutritional and sensory properties via the conversion of slow muscle fibers, the deposition of intramuscular fat [15,16], and the formation of flavor substances [17,18]. In addition, numerous studies had also found that the cage-free rearing system had effects on immune response [9], the microbiome [19] and the blood biochemistry [20] in chickens. However, cage-free mode is a relatively costly rearing method with low growth performance and a low feed conversion ratio. Meanwhile, cage-free rearing mode is not easy to standardize and has some specific risks. For example, poultry in a cage-free rearing system has more opportunities to be exposed to pathogens by the natural environment [21]. Therefore, the choice of rearing system appears to be crucial for chicken meat production and quality.

The Lueyang black-bone chicken is one of the most famous Chinese black-bone chicken breeds and is mainly distributed in Lueyang County, Shaanxi, China, which is located in the Qinling Mountains [22]. With a long history of domestication, Lueyang black-bone chickens have been raised in cage-free rearing systems to adapt to the local geographical environment [23]. Due to the relatively high cost of cage-free rearing, the caged system is adopted for Lueyang black-bone chicken rearing to reduce costs and promote growth performance. Recently, several reports have investigated the effects of different rearing modes on the growth performance and meat quality of Lueyang black-bone chickens. In a previous study, we found that cage-free mode could improve the pH value and tenderness and reduce the hardness by promoting the formation of slow-twitch fibers and intramuscular fat in chicken thigh muscle [23]. In addition, metabolites such as amino acids, fatty acids, and nucleotides affect chicken meat nutrients and flavors. Zhang et al. reported that Lueyang black-bone chickens in the caged group had higher levels of total amino acids and saturated fatty acids than chickens in the cage-free group [24]. Furthermore, they also found that the contents of inosine monophosphate (IMP), glutamate, palmitic acid, and linoleic acid were greater in cage-free chicken breast muscles than in the caged group. However, these previous studies focused only on partial metabolites to explore the effects of different rearing systems on the meat quality of Lueyang black-bone chickens. This study aimed to investigate the effects of caged and cage-free rearing systems on the muscle metabolites and meat quality of Lueyang black-bone chickens using untargeted metabolomic analysis.

## 2. Materials and Methods

### 2.1. Ethics Approval Statement

All animal experimental procedures were approved and conducted in accordance with the guidelines of the Institutional Animal Care and Use Committee of Shaanxi University of Technology (SLGQD/09/2017).

### 2.2. Animal Resources and Rearing Systems

Before the experiment, a total of 600 one-day-old female Lueyang black-bone chicks were raised in cages within the same brood house until they reached 7 weeks of age. Subsequently, 600 hens were isolated and randomly allocated to two groups: caged rearing (CR) or cage-free rearing (CF). Each group contained 3 replicates, with 100 chickens in each replicate. For the CR groups, two chickens were kept in each conventional cage, and every chicken had an average floor area of 0.15 m^2^. The cage system was environmentally controlled, in which the average temperature was 20–25 °C, the relative humidity was 65–70%, and the indoor photoperiod was 16:8 light: dark. For the CF groups, chickens were fed in an indoor floor house (bird/0.15 m^2^) with an adjacent outdoor cage-free area (bird/1 m^2^). The indoor environmental conditions were controlled similarly to those of the CR group, and the outdoor average temperature was 20–25 °C in the local mountain areas during the formal experiment. All chickens had unrestricted access to feed and water, with identical feed formulations and nutrient levels in both groups. After being raised for 20 weeks in the two rearing systems, the chickens were euthanized using carbon dioxide anesthesia followed by conventional neck cutting.

### 2.3. Carcass Yield and Meat Quality Assessment

Fifty hens were randomly selected from each rearing system and were slaughtered after fasting for 12 h. The feathers were pulled out, the viscera were removed, and the carcass yield of the chickens was measured. Then, ten slaughtered chickens were randomly chosen, and the left leg (thigh) muscle (LM) and breast muscle (BM) cuts were isolated and kept at 4 °C for muscle quality assessment, including drip loss, pH_24_, and shear force. For drip loss measurements, fresh muscle samples were cut into 1 cm^3^ pieces and weighed. Then, each sample was independently packaged in an air-filled plastic bag and hung at 4 °C in a refrigerator. After 24 h, the muscle samples were dried and weighed again, and the difference in weights for each sample indicated drip loss [25]. pH_24_ evaluation was conducted by inserting a pH meter (HI8242C, HANNA, Shanghai, China) into the muscle at a depth of 1 cm to determine the pH after 24 h of slaughter. The shear force of the meat samples was measured using a texture analyzer (MT01, Shanghai Bosin, Shanghai, China). Briefly, the muscle samples were bathed in water at 80 °C in sealable bags for half an hour, chilled to room temperature, and then cut into strips with a size of 1.0 cm (width) × 0.5 cm (thickness) × 2.5 cm (length). The strips parallel to the muscle fiber were prepared and sheared vertically [26]. The shear force was expressed in Newtons. Ten replicates were measured for each group, and the average value represented the meat quality traits.

### 2.4. Untargeted Metabolomic Analysis

After slaughter, the right leg (thigh) and breast muscles were isolated from six randomly selected chickens in each group, frozen in liquid nitrogen immediately, and then stored at −80 °C for metabolite and volatile organic compound assays. For the CR groups, the breast and leg muscles were designated CR6_BM and CR6_LM, respectively. The breast and leg muscles in the CF group were designated CF6_BM and CF6_LM, respectively. Six biological replicates were prepared for each treatment for untargeted metabolomic analysis via liquid chromatography–mass spectrometry (LC–MS). For metabolite extraction and quality control, a standard sample production technique was used (Majorbio Biotechnology Company, Shanghai, China). The chromatographic column used was an HSS T3 (2.1 × 100 mm, 1.8 μm). Gradient elution was performed with 5% acetonitrile and 95% water (containing 0.1% formic acid) (A), 47.5% isopropyl alcohol, 47.5% acetonitrile, and 5% water (containing 0.1% formic acid) (B) as the mobile phase. The flow rate was 0.4 mL/min, and the sample size was 2 µL. The temperature of the column was maintained at 40 °C. The mass spectrometric data were collected using a Thermo UHPLC-Q Exactive HF-X system with an electrospray ionization (ESI) source. The experimental parameters were as follows: The ion source temperature was set to 425 °C, and the capillary temperature was set to 325 °C. The normalized collision energy was set to 20–40–60 V rolling. The spray voltage was set to +3500 V and −3500 V. Full MS resolution was 60,000, and MS/MS resolution was 7500. The range of the MS scan was from m/z 70 to 1050. The positive and negative ion scanning modes were used to collect signals. Data acquisition was performed with the data-dependent acquisition (DDA) mode.

Principal component analysis (PCA) and partial least squares discrimination analysis (PLS-DA) in positive and negative ion modes were performed to determine the overall metabolic changes among comparable groups. By comparison, metabolites with variable importance in projection (VIP) > 1 and adjusted *p* < 0.05 were considered significantly differential metabolites (DMs). Subsequently, the screened DMs were analyzed via the Majorbio cloud platform (https://cloud.majorbio.com), which included a Venn diagram and GO and KEGG pathway enrichment analysis.

### 2.5. Comparative Analysis of the Volatile Organic Compounds

The leg and breast muscle samples from the CR and CF groups (4 treatments × 3 biological replicates) were prepared for volatile organic compound analysis. The volatile compounds in black-bone chicken samples were detected using gas chromatography–ion migration spectrometry (GC-IMS) (FlavorSpec, G.A.S., Shandong, China) as previously described [27]. Muscle samples (2.0 g) were placed in 20 mL headspace bottles and incubated at 60 °C for 20 min. Subsequently, 500 μL of head space were injected into the splitless injector by a heated syringe (80 °C) and driven into the MXT-5 chromatic column (15 m × 0.53 mm × 1 μm) by 99.99% nitrogen as the carrier gas and drift gas at a programmed flow as follows: 2 mL/min for 2 min, 10 mL/min for 10 min, 100 mL/min for 15 min, and eventually 150 mL/min for 10 min. The drift gas was set at 150 mL/min. The separated molecules were ionized and moved to a drift tube at a constant temperature (45 °C) and voltage (5 kV). The unique compounds were identified by the retention time in the GC part and the drift time in the IMS part with NIST Library and IMS database retrieval software (VOCal Software, 0.4.03) from G.A.S. IMS data were analyzed by instrumental analysis software, including LAV (from G.A.S., Dortmund, Germany, version 2.0.0), Reporter, and GC × IMS Library Search. PCA was performed for the clustering of samples. A gallery plot of the identified flavor substances was constructed to better analyze the differences in flavor substances between the CR and CF groups.

### 2.6. Correlation Analysis of Metabolites and Volatile Organic Compounds

The correlation between DMs and flavor substances was assessed and dynamic correlation heatmaps were generated by the O2PLS OmicShare website tool (https://www.omicshare.com/tools/Home/Soft/getsoft (15 March 2024)).

### 2.7. Data Analysis

All the data represent at least three independent experiments, and the quantitative results are presented as the means ± S.D.s. Differences between groups were assessed by using SPSS v23 software via one-way or two-way ANOVA, and *p* < 0.05 was considered to indicate statistical significance.

## 3. Results and Discussion

### 3.1. Chicken Carcass Yield and Meat Quality under Different Rearing Systems

Carcass traits and the meat quality in Lueyang black-bone chickens are affected by the rearing system. After being reared for 20 weeks, the carcass yield of hens in the CR group (1.52 ± 0.15 kg, *n* = 50) was greater than that in the CF group (1.26 ± 0.09 kg, *n* = 50). Moreover, the shear force of the leg muscles in the cage-free group was significantly greater than that in the caged group, but there was no significant difference in the shear force of the breast muscles (Table 1). However, the different rearing systems did not significantly affect the pH or drip loss in the leg or breast muscles of Lueyang black-bone chickens (Table 1).

### 3.2. Untargeted Metabolomics and Differential Metabolite Analysis

#### 3.2.1. Quality Control of the Metabolomes of Muscle Samples

**Figure 1:**
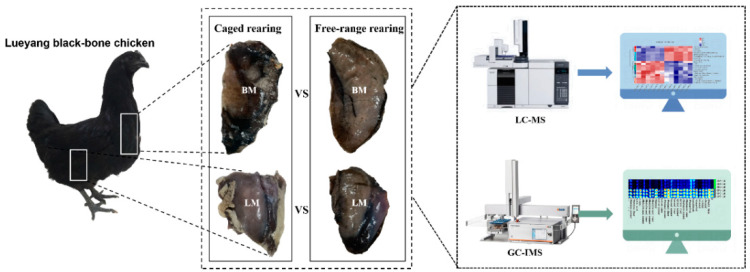
Overview of the experimental design. BM: breast muscle. LM: leg muscle. LC-MS: liquid chromatograph–mass spectrometer. GC-IMS: gas chromatography–ion migration spectrometry.

**Figure 2:**
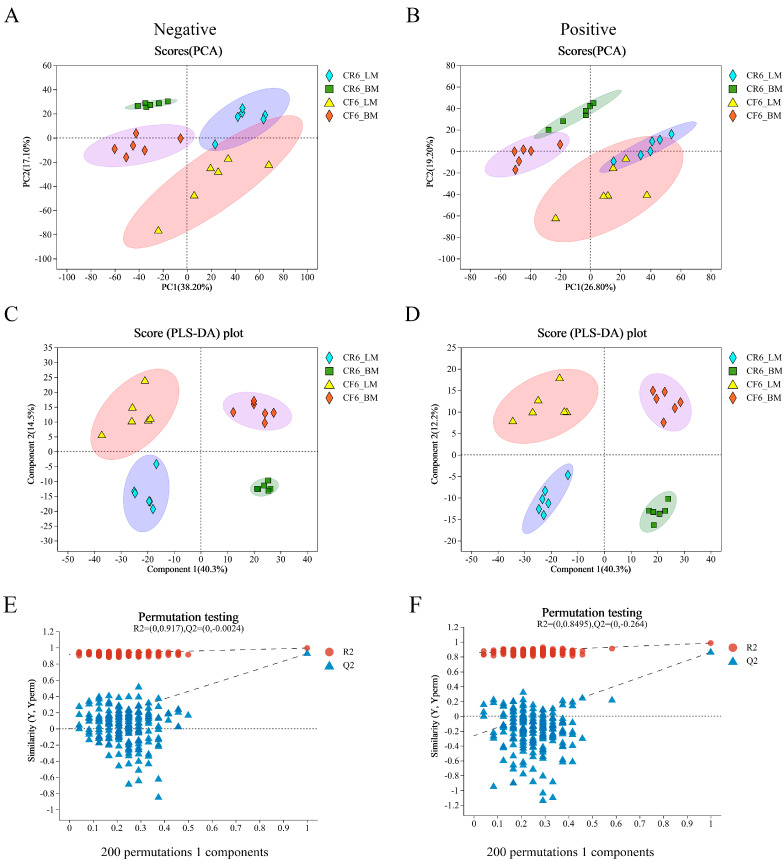
Multivariate analysis of metabolomics data from different chicken samples. (**A**,**B**) PCA score plots based on metabolites from negative and positive ion modes; (**C**,**D**) PLS-DA score plots based on metabolites from negative and positive ion modes. (**E**,**F**) Permutation tests were used to assess the accuracy of the PLS-DA models in negative and positive ion modes.

The breast and leg muscles were isolated from the two differently reared chickens for untargeted metabolomic analysis (Figure 1). The metabolomics data from the four groups were analyzed through PCA and PLS-DA in negative and positive ion modes to assess the differences in metabolites from intergroup samples. The PCA and PLS-DA results showed that 24 samples were tightly clustered into their corresponding groups. However, the samples of leg muscles partially overlapped between CR and CF in PCA, especially in positive ion mode (Figure 2A,B). PLS-DA revealed significant differences in metabolites among the four groups (Figure 2C,D). Subsequently, permutation tests were conducted to prevent overfitting of the model. The intercept of Q2 and the *y*-axis was less than 0, indicating a low risk of overfitting these models (Figure 2E,F). The quality control analysis suggested that the quality of the metabolome assay data was sufficient for subsequent metabolite expression analysis.

#### 3.2.2. Analysis of All Metabolites

**Figure 3:**
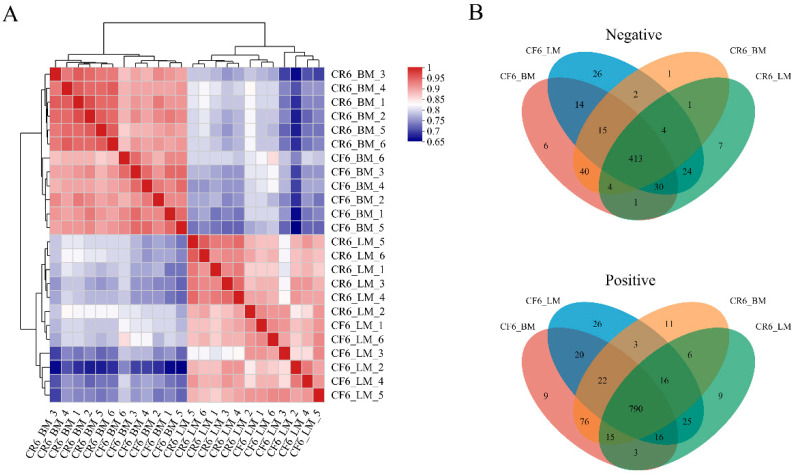
Analysis of metabolites among the four groups. (**A**) Heatmap of hierarchical clustering of differentiated metabolites among the four groups. (**B**) Venn analysis of shared metabolites in negative and positive ion modes.

A total of 1635 metabolites were screened in breast and leg muscles from CR and CF, including 588 and 1047 metabolites in negative and positive ion modes, respectively. Based on the metabolites, heatmap analysis revealed significant differences among the four groups and good clustering in the samples of each group (Figure 3A). A total of 1203 shared metabolites were identified in the four groups, including 413 and 790 metabolites in the negative and positive ion modes, respectively (Figure 3B). The shared metabolites illustrated that the skeletal muscles of the caged and cage-free rearing modes had similar metabolic pathways.

#### 3.2.3. Screening and Correlation Analysis of Differential Metabolites

**Figure 4:**
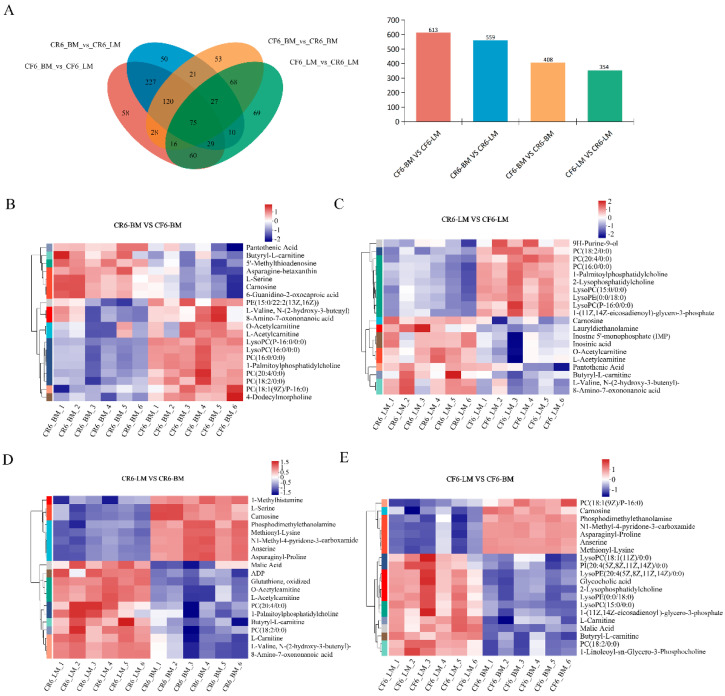
Analysis of DMs in skeletal muscles. (**A**) Venn diagram of the total DM analysis. Heatmap of hierarchical clustering of DMs in the breast and leg muscles between cage-free and caged chickens. (**B**–**E**) The top 20 DMs in the leg and breast muscles.

The screening criteria used to select DMs in the breast and leg muscles between the caged and cage-free groups were VIP > 1 and *p* value < 0.05. A total of 911 DMs among the four groups were identified (Figure 4A). Among the breast muscles in the CF and CR groups, 408 DMs (CF6_BM vs. CR6_BM) were identified (Figure 4A); 284 DMs were upregulated, including PC (18:2/0:0), LysoPC (16:0/0:0), and acetylcarnitine; and 124 DMs were downregulated, including pantothenic acid, 2-hydroxystearic acid, L-serine, and carnosine (Figure 4B and Figure S1A). For leg muscles between the CF and CR groups, 235 metabolites were upregulated, including LysoPE (P-18:0/0:0), LysoPC (15:0/0:0), and PC (16:0/0:0), and 119 metabolites were downregulated, including carnosine, acetylcarnitine, and pantothenic acid (Figure 4C and Figure S1B). Lysophospholipids play anti-inflammatory, anti-hemostatic, and anti-oxidant roles and reduced the fat deposition in the blood [28]. The main role of acetylcarnitine is to transport fatty acids into the mitochondrial matrix where fatty acid metabolism occurs [29]. In addition, carnosine has many physiological roles, including pH buffering, antioxidant capacity, and the capacity to protect against the formation of advanced glycation and lipoxidation end-products [30]. Pantothenic acid is involved in the metabolism of carbohydrates, lipids, and proteins. A previous study demonstrated that pantothenic acid deficiency could cause duck growth retardation, poor feathering, dermatosis, and high mortality [31].

The metabolites in the breast and leg muscles greatly differed under the same rearing mode. In the caged rearing system, 559 DMs (CR6_LM vs. CR6_BM) were identified between the breast and leg muscles (Figure 4A), among which 254 DMs, including anserine, L-serine, and carnosine, were upregulated and 305 DMs, including acetylcarnitine, PC (18:2/0:0), and ADP, were downregulated (Figure 4D and Figure S1C). In the cage-free mode, 613 DMs (CF6_LM vs. CF6_BM) were screened between the breast and leg muscles (Figure 4A), among which 306 metabolites, including anserine, carnosine, and methionyl-lysine, were upregulated, and 307 metabolites inducing LysoPC (15:0/0:0), L-serine, and 2-lysophosphatidylcholine, were downregulated (Figure 4E and Figure S1D). Though the meat traits (pH, shear force, and drip loss) showed no significant difference between leg and breast muscles in the same rearing system (Table 1), the metabolites were dramatically different between the two muscle types, which may affect chicken nutritional values and flavors.

#### 3.2.4. Analysis of Differential Metabolite Clusters

**Figure 5:**
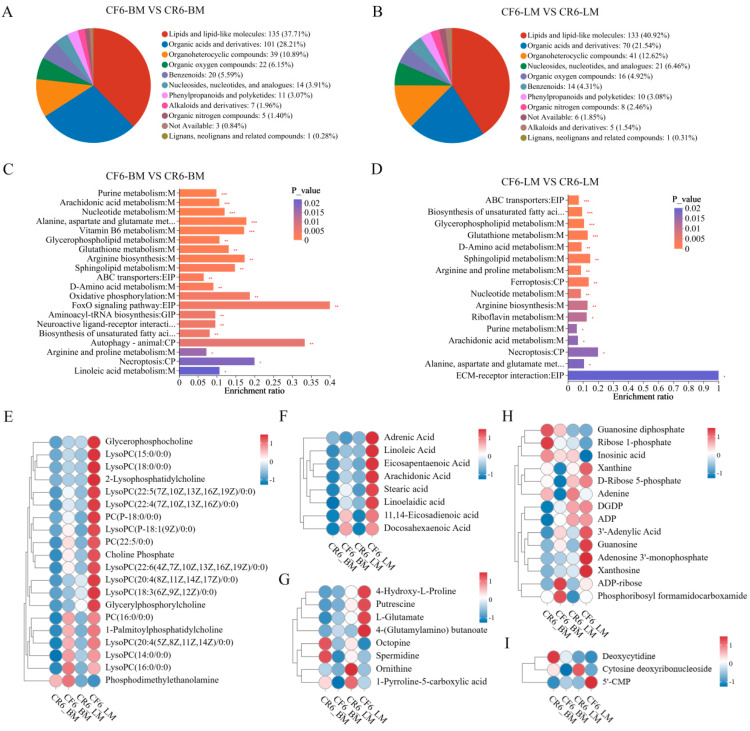
Cluster analysis of DMs in the breast and leg muscles between the caged and cage-free groups. HMDB compound classification of DMs in breast muscles (**A**) and leg muscles (**B**). KEGG enrichment analysis of DMs in breast muscles (**C**) and leg muscles (**D**). Hierarchical clustering analysis of DMs involved in glycerophospholipid metabolism (**E**), biosynthesis of unsaturated fatty acids (**F**), arginine and proline metabolism (**G**), purine metabolism (**H**), and pyrimidine metabolism (**I**). * *p* < 0.05; ** *p* < 0.01; *** *p* < 0.001.

To investigate the effect of the rearing system on chicken muscle metabolism, the DMs of the leg and breast muscles in the CR and CF groups were further analyzed. The DMs were clustered into 11 different categories, including lipids and lipid-like molecules, organic acids and derivatives, and organoheterocyclic compounds (Figure 5A,B). The DMs were predominantly involved in metabolic pathways, especially lipid metabolism, amino acid metabolism, nucleotide metabolism, and carbohydrate metabolism (Figure S2). The top class of DMs was lipid metabolism, and more than 60 lipids were detected in the leg and breast muscles. In comparison, different lipids in the breast and leg were dominated by glycerophosphocholines, fatty acids, and conjugates (Figure S3).

KEGG enrichment analyses were used to further evaluate the molecular function of DMs in Lueyang black-bone chickens. KEGG enrichment analyses demonstrated that the DMs between the two rearing systems were predominantly enriched in the following metabolic pathways: glycerophospholipid metabolism, biosynthesis of unsaturated fatty acids, arginine and proline metabolism, purine metabolism, and pyrimidine metabolism (Figure 5C,D).

For glycerophospholipid metabolism, 20 phospholipids and their derivatives were identified (Figure 5E). Compared with those in the CR group, the concentrations of 19 phospholipids, mainly phosphatidylcholines (such as PC (P-18:0/0:0), PC (16:0/0:0), and PC (22:5/0:0)) and lysophosphatidylcholines (such as LysoPC (20:4(5Z,8Z,11Z,14Z)/0:0), LysoPC (16:0/0:0), and LysoPC (14:0/0:0)), were significantly greater in the CF group. For the biosynthesis of unsaturated fatty acids, 11,14-eicosadienoic acid, linoleic acid, docosahexaenoic acid, eicosapentaenoic acid, stearic acid, and arachidonic acid were significantly upregulated in both the breast and the leg muscles of the CF group, while linolenic acid and adrenic acid were significantly increased only in the leg muscles of the CF group (Figure 5F).

The hierarchical clustering analysis of eight representative metabolites involved in arginine and proline metabolism is shown in Figure 5G. These results demonstrated that the concentrations of 4-hydroxy-L-proline, putrescine, 4-(glutamylamino) butanoate, and L-glutamate were significantly upregulated, while the expression levels of octopine, spermidine, ornithine, and 1-pyrroline-5-carboxylic acid were decreased in the breast and leg muscles from the CF group. These results suggest that the accumulation of 4-hydroxy-L-proline and L-glutamate was increased and that the production of spermidine and ornithine was decreased in chicken meat from the CF group.

In addition, 14 significant DMs involved in purine metabolism were identified (Figure 5H). In the breast muscles, the concentrations of 3′-adenylic acid and ADP increased, and the abundances of xanthine, adenine, guanosine diphosphate (GDP), and ribose 1-phosphate decreased in the CF group compared with those in the CR group. In the leg muscles, the concentrations of 3′-adenylic acid (adenosine 3′-monophosphate, 3′-AMP), guanosine, and xanthosine were increased, while the levels of ADP ribose and inosinic acid (inosine monophosphate, IMP) were decreased in the CF group compared with those in the CR group. With respect to pyrimidine metabolism, the levels of deoxycytidine and cytosine deoxyribonucleoside were lower in the breast and leg muscles of the CF group than in those of the CR group, but the amount of 5′-CMP was significantly greater in the leg muscles of the CF group (Figure 5I). These results indicate that the cage-free rearing system increased the abundance of purine and pyrimidine metabolites, which largely contributed to the improvement in meat flavor.

### 3.3. Analysis of the Volatile Organic Compounds in Chicken Meat

Volatile organic compounds were identified by GC-IMS in the leg and breast muscles of CR and CF chickens. Based on the data, the PCA results showed high reproducibility of the inner group samples, which were obviously separated among the groups (Figure S4). Forty-seven flavor substances were identified in the muscles, including 14 aldehydes, 9 alcohols, 10 ketones, 2 esters, 1 acid, 1 furan, and 10 unknown substances. The top three compounds were ketones, alcohols, and aldehydes (Table S1).

**Figure 6:**
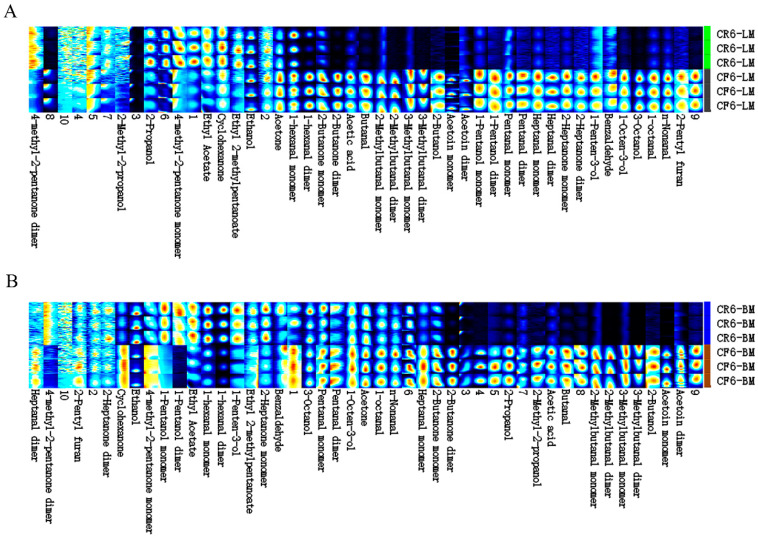
Analysis of volatile organic compounds in leg muscles (**A**) and breast muscles (**B**) between the caged and cage-free modes. Colors represent the concentration of substances; deep blue indicates background; light blue indicates low concentration; yellow indicates high concentration; red indicates higher concentration. The darker the color, the higher the concentration.

In the leg muscles, the contents of 32 flavor substances were significantly greater in CF chickens, including 14 aldehydes (2-methylbutanal, 3-methylbutana, heptanal with fruity aroma, etc.), 7 ketones (2-heptanone with a pear scent and acetoin dimer with a milky scent, etc.), 6 alcohols (1-octen-3-ol with mushroom odor, 2-butanol with wine odor, 1-penten-3-ol with fruity odor, etc.), and 1 furan (2-pentyl furan with fruit fragrance) (Figure 6A). In addition, 12 flavor compounds were more abundant in CR chickens, including two alcohols (2-methyl-2-propanol with a camphor odor and 2-propanol with mixed ethanol and acetone), three ketones (cyclohexanone with a mint odor and 4-methyl-2-pentanone with a camphor odor), and two esters (ethyl acetate and ethyl 2-methylpentanoate with a fruity flavor) (Figure 6A). The contents of ethanol and two unknown substances were not significantly different between the two rearing modes.

In the breast muscles, the levels of 32 volatile substances, including 12 aldehydes (1-octanal, 2-methylbutanal, butanal, etc.), 6 ketones (cyclohexanone, 2-butanone, acetoin, etc.), 5 alcohols (2-propanol, 1-octen-3-ol, 1-pentanol dimer, etc.), and 1 acid (acetic acid), were significantly upregulated in the CF chickens (Figure 6B). Additionally, 11 flavor substances, including four alcohols (ethyl acetate, 1-penten-3-ol, 3-octanol, and ethanol), two aldehydes (1-hexanal dimer and monomer), three ketones (4-methyl-2-pentanone and 2-heptanone monomer), and two esters (ethyl 2-methylpentanoate and ethyl acetate), were more abundant in CR chickens (Figure 6B). Moreover, the differences in 2-pentyl furan, 2-heptanone dimer, and two unknown substances were not significant between the two rearing modes.

### 3.4. Correlations between Differential Metabolites and Volatile Organic Compounds

**Figure 7:**
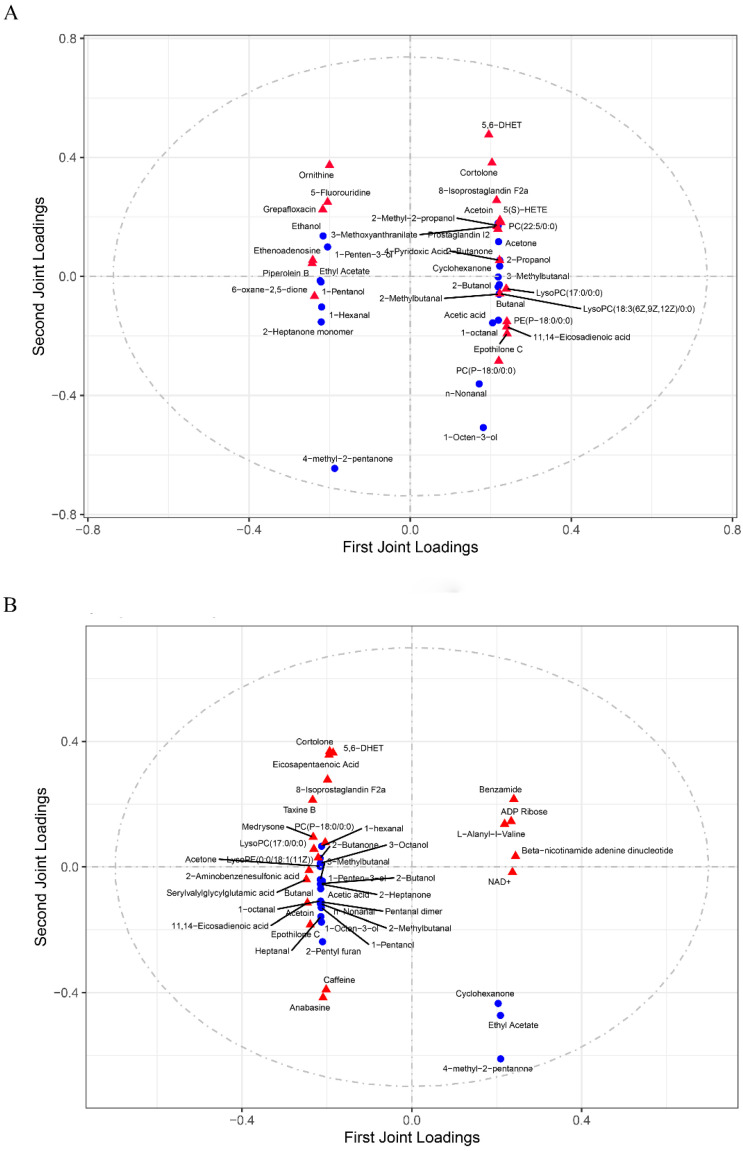
Correlations between the DMs and volatile organic compounds in the breast muscles (**A**) and leg muscles (**B**) of the caged and cage-free rearing modes.

Comparative analysis was performed to determine the relationships between DMs and volatile organic compounds in chicken meat. For the breast muscles, 20 flavor substances, including 2-butanol, 3-methylbutanal, and acetoin, were positively correlated with 57 DMs, including 5(S)-HETE, prostaglandin I2, PC (P-18:0/0:0), LysoPC (17:0/0:0), and 11,14-eicosadienoic acid, and these DMs had high content in the muscles from CF chickens (Figure 7A and Figure S5A). These 20 flavor compounds were mainly negatively correlated with 10 DMs, including piperolein B, ethenoadenosine, 6-oxane-2, and 5-dione, and these DMs were less abundant in CF chicken muscles. Additionally, 10 flavor substances, including ethanol, ethyl acetate, and 2-heptanone monomer, were mainly positively correlated with 10 metabolites, such as ornithine, piperolein B, and 5-fluorouridine, and these metabolites were more abundant in CR chicken muscles. Ten flavor compounds were negatively correlated with 57 DMs, such as 5,6-DHET, 8-isoprostaglandin F2a, and LysoPC (17:0/0:0), and these metabolic pathways were less abundant in muscles from CR.

Correlation analysis between metabolites and flavor compounds in leg muscles under the two rearing modes revealed that 29 flavor substances, including 1-octen-3-ol, 3-methylbutana, and 2-heptanone, were mainly positively correlated with 37 DMs, such as PC (P-18:0/0:0), LysoPC (17:0/0:0), and 11,14-eicosadienoic acid (Figure 7B and Figure S5B), and the levels of these DMs were greater in muscles from CF chickens. In addition, 30 flavor substances were negatively correlated with 11 DMs, including ADP ribose, NAD+, and benzamide, and these DMs were less abundant in CF chicken muscles. In addition, four flavor compounds, such as 4-methyl-2-pentanone, ethyl acetate, and cyclohexanone, were mainly positively correlated with nine DMs, such as ADP ribose, NAD+, and benzamide, and these DMs were more abundant in muscles from CR. Flavor compounds were mainly negatively correlated with 44 metabolites, such as 5,6-DHET, cortolone, and 8-isoprostaglandin F2a, and these metabolites were less abundant in muscles from CR chickens.

Among these metabolites, 5(S)-HETE and prostaglandin I2 are involved in arachidonic acid metabolism, PC (P-18:0/0:0) and LysoPC (17:0/0:0) are involved in glycerol phosphate metabolism, and 11,14-eicosadienoic acid is involved in unsaturated fatty acid metabolism (Figure 5F). These metabolites had relatively high levels in muscles from CF chickens. These results suggest that high contents of lipids largely contributed to increased amounts of flavor substances in the muscles of CF chickens.

## 4. Discussion

Caged and cage-free rearing patterns significantly affected chicken growth and meat quality. Consistent with the findings of a previous study [11], we found that the carcass yield of CF chickens was lower than that of CR chickens. Due to more opportunities for physical exercise and energy consumption, the cage-free rearing system had a negative effect on body weight gain compared with the cage-rearing system [26]. In addition, pH, a vital characteristic of meat quality, is closely related to the amount of muscle glycogen, which decomposes into lactic acid after slaughter [32]. We found that the pH of the leg and breast muscles after 24 h of slaughter was not significantly different between the two rearing modes. However, Zhang et al. reported that the pH of breast muscles in cage-free Lueyang black-bone chickens was significantly greater than that in caged chickens after 1 h of slaughter [24]. This difference is possibly due to different post-slaughter measurement time. Shear force and drip loss directly affect muscle juiciness and tenderness [33]. In the present study, compared to that in the caged group, only the shear force of the leg muscles was significantly greater in the cage-free group. Jin et al. reported that the shear force was greater and the drip loss was lower in the breast and thigh muscles of free-range Wannan Yellow chickens [11]. Physical activity could enhance the development and density of chicken muscle fibers, and this might be a major factor in increasing shear force and decreasing drip loss.

Biomacromolecules, including lipids, proteins, carbohydrates and nucleotides, are the main compounds of muscles, and their metabolism is largely impacted by rearing systems. In our study, a large number of differential metabolites were mainly involved in lipid metabolism, amino acid metabolism, nucleotide metabolism, etc. Regarding lipid metabolism, we found that a large number of phospholipids were more abundant in CF chickens than in CR chickens. A previous study reported that phospholipids, particularly PCs, have essential functions, including cell membrane formation, cholesterol homeostasis, and triacylglycerol storage and secretion [34]. LysoPC has anti-inflammatory, anti-hemostatic, and cytotoxic effects [28]. We also found that unsaturated fatty acids, such as 11,14-eicosadienoic acid, linoleic acid, docosahexaenoic acid, eicosapentaenoic acid, and arachidonic acid, participated in unsaturated fatty acid metabolism, linoleic acid metabolism, and arachidonic acid metabolism. In addition, the lipolytic effect of phospholipids largely contributes to the increase in free fatty acids [35]. Fatty acid oxidation products can greatly enhance the formation of flavor compounds and aroma compounds [36,37]. These data demonstrate that chicken meat in the cage-free groups had better nutritional and flavor characteristics.

Moreover, there were significant differences in the metabolism of arginine and proline in muscles between the two rearing modes. We found that 4-hydroxy-L-proline and L-glutamate were upregulated, while ornithine and 1-pyrroline-5-carboxylic acid were downregulated in the breast and leg muscles of the CF group compared with those of the CR group. Hydroxy-L-proline is a unique amino acid derived from proline. Proline is synthesized from glutamate and arginine, and proline residues can be hydroxylated to form 4-hydroxy-L-proline in the endoplasmic reticulum [38]. Previous studies reported that 4-hydroxy-L-proline not only is a main component of collagen [39] but also scavenges oxidants and regulates the redox state of cells [40]. Pyrroline-5-carboxylic acid is an intermediate product in the synthesis of proline and is also converted into ornithine by ornithine-aminotransferase. Furthermore, arginine can be decomposed to ornithine and urea by arginine kinase. Ornithine is converted into putrescine, glutamine, and proline through a series of reactions in which putrescine can produce spermidine and spermine, which are collectively known as polyamines. Polyamines are involved in the biological metabolism of DNA, RNA, and proteins and play important roles in the growth, proliferation, and differentiation of cells [41]. In addition to being involved in the formation of proline, L-glutamate plays an important role in multiple protein metabolism processes and is involved in many important chemical reactions in animals [42]. L-glutamate is also an important flavor substance that can improve the composition of muscle fatty acids [41].

In addition to protein and lipid metabolism, nucleotide metabolism also influences meat quality, especially meat flavor [43]. IMP and AMP greatly contribute to the sweet and umami taste of chicken meat, respectively [44]. AMP and IMP are progressively changed to inosine and hypoxanthine in fresh meat within a few hours after slaughter [45]. In this study, the AMP content was significantly greater in the breast and leg muscles of cage-free chickens than in those of caged chickens. The IMP content decreased significantly in the leg muscles of cage-free chickens. However, previous studies demonstrated that the content of IMP was greater in cage-free chicken muscles than in caged chicken muscles [24,46], which is contrary to the results of the present study. This phenomenon could be explained by the conversion of IMP to AMP in chicken leg muscles under the cage-free rearing mode.

The contents of volatile substances are the key factors affecting the overall flavor of meat. The formation of volatile components, including ketones, aldehydes, alcohols, esters, acids, and furans, is related to amino acid degradation, unsaturated fatty acid oxidation, and carbohydrate metabolism [47,48]. In this study, ketones with low odor thresholds and pleasant odors, mainly acetoin dimers and monomers, which play important roles in sensory characteristics, had extremely high contents in the breast and leg muscles of Lueyang black-bone chickens. Additionally, most aldehydes have a fruity aroma with a low odor threshold [49]. Aldehydes might be produced by the oxidation of fatty acids and amino acid degradation. For instance, nonanal and hexanal are produced by the oxidation of polyunsaturated fatty acids [47]. Hexanal can also be formed from amino acids and free acids as precursors [50]. Fourt4een and 12 aldehydes were more abundant in the leg and breast muscles of CF chickens, respectively, which indicates that aldehydes greatly contributed to the strong aroma of CF chicken muscles.

Moreover, most alcohols have a high odor threshold, which contributes less to the flavor of meat but has synergistic effects on the overall flavor. However, 1-octen-3-ol, an unsaturated alcohol, is the dominant flavor, and it has a low odor threshold and mushroom and citrus odors. 1-Octen-3-ol can be produced through the degradation of linoleic and linolenic acids [51]. Compared to those in muscles from CR chickens, the relative content of 1-octen-3-ol was significantly greater and had a positive effect on the flavor of CF chicken muscles. In addition, acids are produced by fatty oxidation or fat hydrolysis into short-chain fatty acids. With a high odor threshold in meat, acid provided a small contribution to the overall flavor of the muscles. Esters were mainly formed by the esterification reaction between alcohols and free fatty acids. Esters have a sweet fruit odor and contribute less to the flavor of meat. We found that the ethyl 2-methylpentanoate and ethyl acetate concentrations were greater in muscles from CR chickens than in those from CF chickens. Furans are produced by Strecker degradation and the Maillard reaction [52]. 2-Pentylfuran has a fruit and vegetable aroma and a low odor threshold and is highly abundant in the leg muscles of CF chickens. In summary, the types and contents of volatile compounds in muscles from CF chickens were significantly greater than those from CR chickens.

## 5. Conclusions

This is an original study on the differences in the meat quality, metabolites, and related pathways of the breast and leg muscles of Lueyang black-bone chickens between caged and cage-free rearing systems. In summary, compared with caged rearing, cage-free rearing significantly reduced chicken carcass yield and increased the shear force of leg muscles. The metabolomic analysis suggests that the cage-free rearing system promoted the accumulation of phospholipids, unsaturated fatty acids, 4-hydroxy-L-proline, L-glutamate, AMP, xanthosine, guanosine, and 5′-CMP in the breast and leg muscles. Moreover, volatile compounds such as aldehydes, ketones, and alcohols were more abundant in the cage-free groups, which contributed significantly to the flavor of the chicken meat. However, further studies are needed to investigate the absolute quantity of nutrients and sensory evaluation of cooked Lueyang black-bone chicken meat between the two rearing systems. Overall, the cage-free rearing mode facilitated the accumulation of nutrients and flavor substances in the chicken meat and was a better rearing system for Lueyang black-bone chickens.

## Figures and Tables

**Table 1:** Comparison of meat quality between different rearing modes and muscle types.

Treatments		Traits (*n* = 10)	
	pH (24 h)	Shear Force (N)	Drip Loss (%)
CR6_LM	5.58 ± 0.27 ^a^	24.15 ± 1.93 ^b^	2.30% ± 0.43% ^a^
CR6_BM	5.60 ± 0.21 ^a^	27.79 ± 1.9 ^ab^	2.77% ± 0.41% ^a^
CF6_LM	5.80 ± 0.28 ^a^	27.98 ± 2.43 ^a^	2.24% ± 0.41% ^a^
CF6_BM	5.91 ± 0.14 ^a^	31.39 ± 2.08 ^a^	2.30% ± 0.43% ^a^

CR6_LM: leg muscle from caged rearing. CR6_BM: breast muscle from caged rearing. CF6_LM: leg muscle from cage-free rearing. CR6_BM: breast muscle from cage-free rearing. Ten replicates were measured for each group. Within the same line for each factor, different lowercase letters (a,b) indicate significant differences (*p* < 0.05).

## Data Availability

Data are contained within the article.

## Supplementary Materials

The following supporting information can be downloaded at: https://www.mdpi.com/article/10.3390/ani14142041/s1. Figure S1: the volcano plots illustration of up and down-regulated DMs in breast and leg muscles between caged and free-range chickens. Figure S2: KEGG pathway classification of DMs in breast muscles (A) and leg muscles (B) between free-range and caged rearing. Figure S3: KEGG pathway classification of lipids in breast muscles (A) and leg muscles (B) between free-range and caged rearing. Figure S4: PCA score plots based on volatile organic compounds from breast muscles (A) and leg muscles (B) between free-range and caged rearing. Figure S5: correlation heatmaps between DMs and volatile organic compounds in breast muscles (A) and leg muscle muscles (B) from free-range and caged chickens. Table S1: the content of volatile organic compounds in breast and leg muscles between caged and free-range modes.
