# Supplementary material for: Identification of Metabolites in Muscles of Lueyang Black-Bone Chickens: A Comparative Analysis of Caged and Cage-Free Rearing Modes Using Untargeted Metabolomic Techniques
Source: Animals (Basel). 2024 Jul 12;14(14):2041. doi: 10.3390/ani14142041 (PMC11274139; doi:10.3390/ani14142041)
Supplement: Supplementary file 1 [file animals-14-02041-s001.zip › Supplementary information.pdf]

Supplementary information

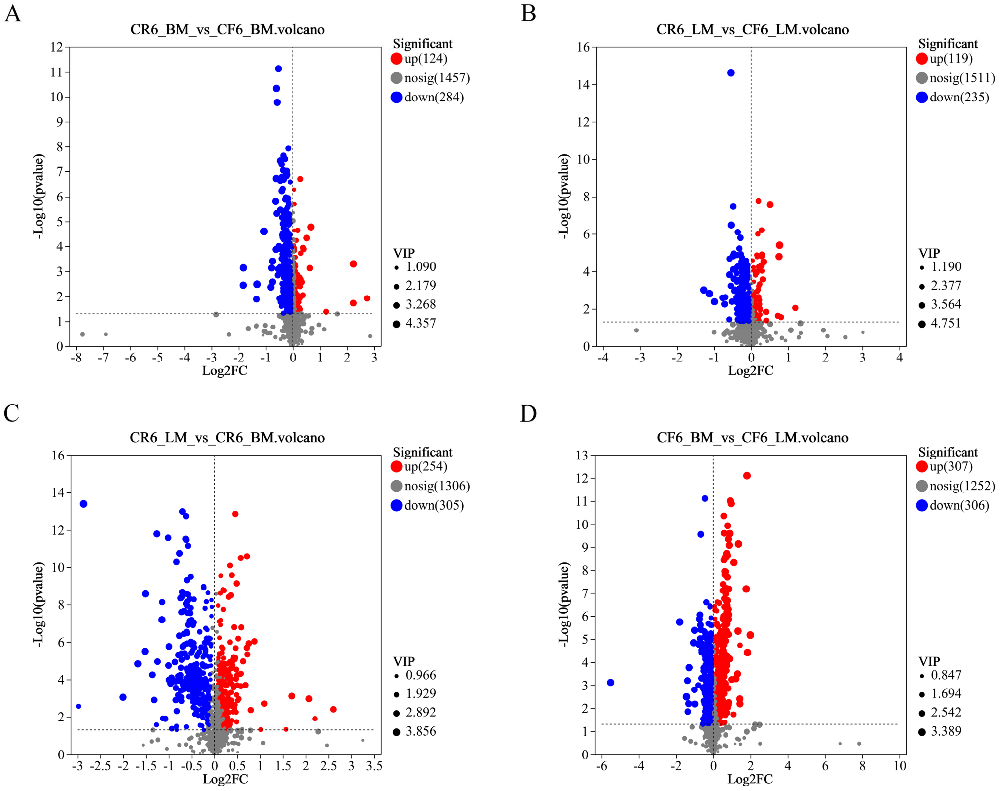

Figure S1. The volcano plots illustration of up and down-regulated DMs in breast and leg muscles between caged and free-range chickens.

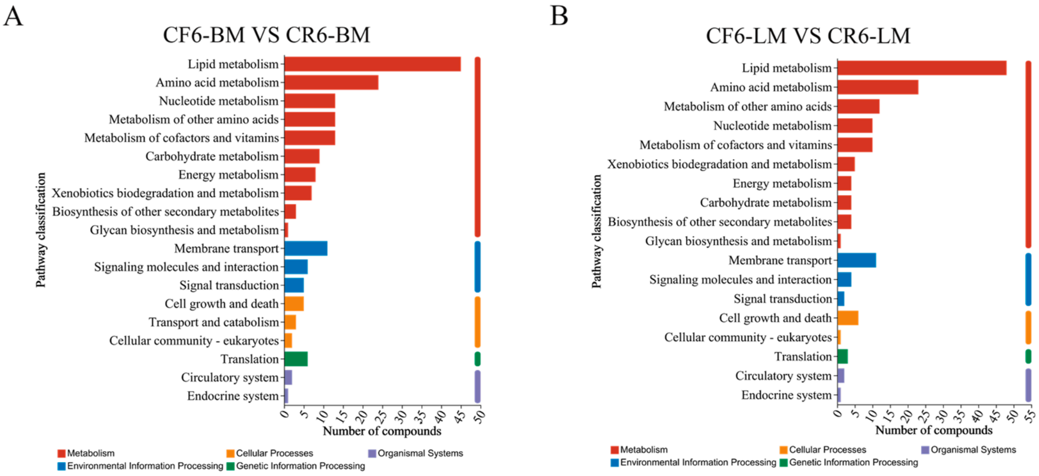

Figure S2. KEGG pathway classification of DMs in breast muscles (A) and leg muscles (B) between free-range and caged rearing.

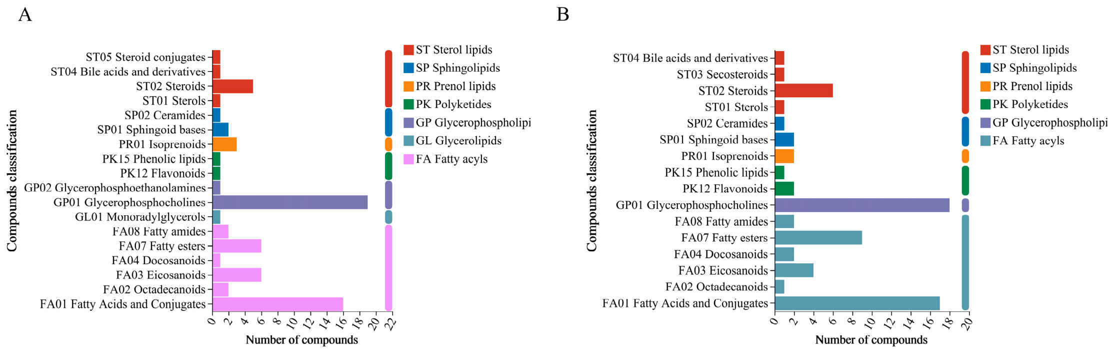

Figure S3. KEGG pathway classification of lipids in breast muscles (A) and leg muscles (B) between free-range and caged rearing.

A

B

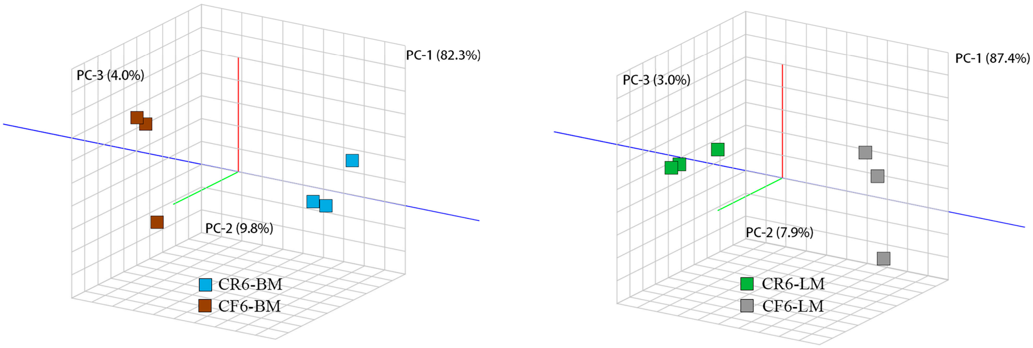

Figure S4. PCA score plots based on volatile organic compounds from breast muscles (A) and leg muscles (B) between free-range and caged rearing.

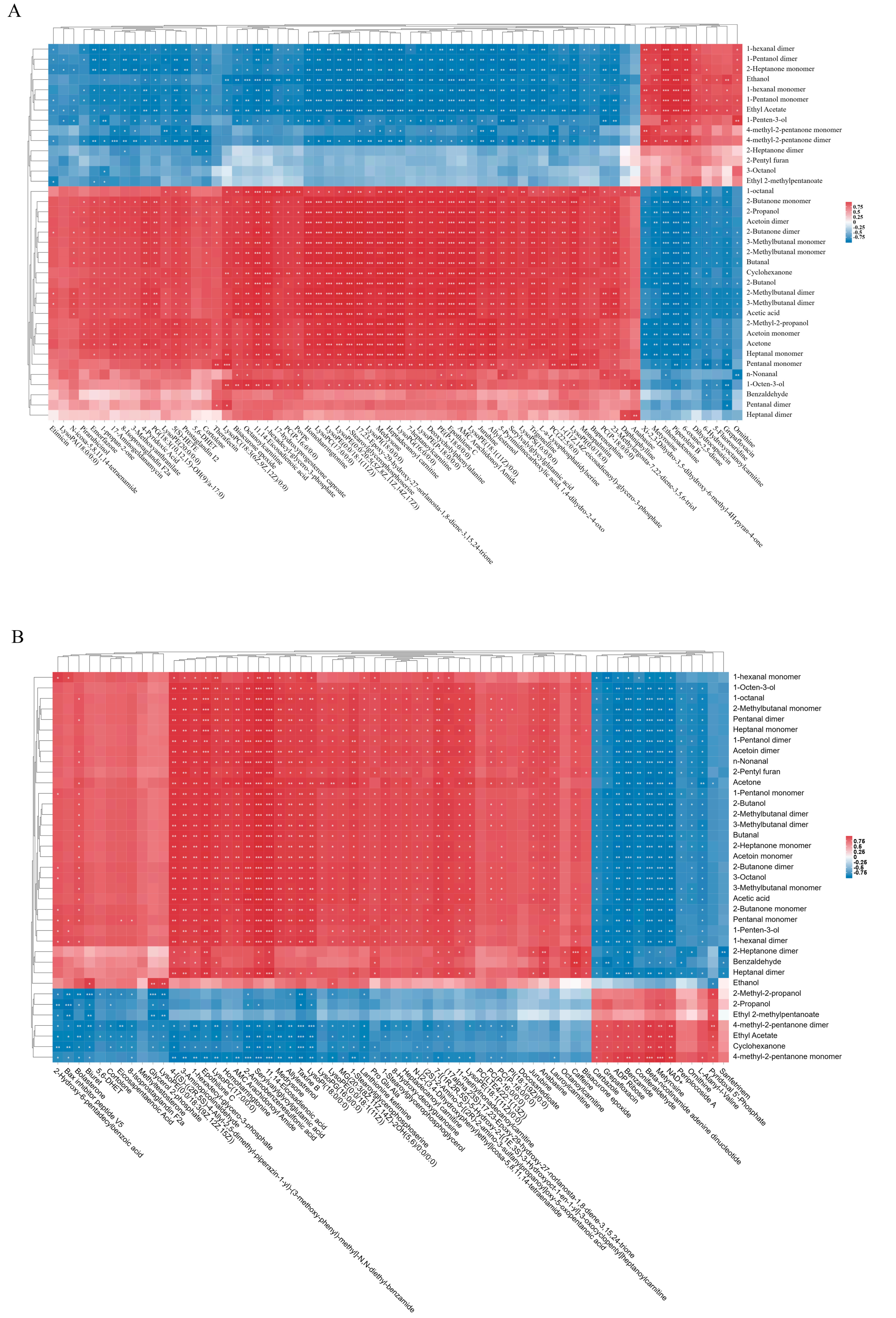

Figure S5. Correlation heatmaps between DMs and volatile organic compounds in breast muscles (A) and leg muscle muscles (B) from free-range and caged chickens.
